# NCMHap: a novel method for haplotype reconstruction based on Neutrosophic c-means clustering

**DOI:** 10.1186/s12859-020-03775-0

**Published:** 2020-10-22

**Authors:** Fatemeh Zamani, Mohammad Hossein Olyaee, Alireza Khanteymoori

**Affiliations:** 1 Department of Computer Engineering, University of Zanjan, Zanjan, Iran; 2 Department of Computer Engineering, Faculty of Engineering, University of Gonabad, Gonabad, Iran

**Keywords:** Bioinformatics, Haplotype assembly, Minimum error correction, Neutrosophic c-means clustering

## Abstract

**Background:**

Single individual haplotype problem refers to reconstructing haplotypes of an individual based on several input fragments sequenced from a specified chromosome. Solving this problem is an important task in computational biology and has many applications in the pharmaceutical industry, clinical decision-making, and genetic diseases. It is known that solving the problem is NP-hard. Although several methods have been proposed to solve the problem, it is found that most of them have low performances in dealing with noisy input fragments. Therefore, proposing a method which is accurate and scalable, is a challenging task.

**Results:**

In this paper, we introduced a method, named NCMHap, which utilizes the Neutrosophic c-means (NCM) clustering algorithm. The NCM algorithm can effectively detect the noise and outliers in the input data. In addition, it can reduce their effects in the clustering process. The proposed method has been evaluated by several benchmark datasets. Comparing with existing methods indicates when NCM is tuned by suitable parameters, the results are encouraging. In particular, when the amount of noise increases, it outperforms the comparing methods.

**Conclusion:**

The proposed method is validated using simulated and real datasets. The achieved results recommend the application of NCMHap on the datasets which involve the fragments with a huge amount of gaps and noise.

## Background

It has been revealed that the human genome shows some degrees of inter-individual and inter-population variations which make it an appropriate target to rigorous functional genomic analysis [1, 2]. Recent cost-effective next-generation sequencing (NGS) technologies have provided a huge amount of genome sequences of individual human [3]. It has been discovered that more than 99% of human genomes are completely identical. Therefore, it turns out that the vast differences among people can be emerged from less than 1% variations [4, 5]. Single nucleotide polymorphisms (SNPs) refer to the genetic variations which are more frequent. A sequence of SNPs that co-occur in a specific chromosome is named as haplotype. In diploid species like humans, there are two copies of each chromosome. Since each haplotype is derived from a copy of a specific chromosome, as a result, there are two copies of haplotypes.

Haplotypes provide more attainable information than individual SNPs which can be remarkable for investigating the relationship between genetic variations and complex diseases [6], studying human history [7], providing personalized medicine [8] and studying biological mechanisms [9].

Although obtaining the haplotypes is an important task, direct experimental analysis of haplotypes is labor-intensive, expensive, and restricted to obtaining local haplotypes. In practice, human haplotypes are provided as sequencing reads (fragments). Assuming the importance of detecting genetic variations accompanied by limitations over molecular approaches, obtaining haplotype information from these numerous fragments may have profound effects on different aspects of medicine and molecular biology [10–13]. Availability of the fragments makes it possible to assemble haplotypes in a process referred to as single individual haplotyping (SIH) [14] which is performed by in silico (computer-aided) analysis using statistical and computational approaches.

For this purpose, the requested region of the specified chromosome is sequenced several times and a number of fragments are provided. Due to the limitations of sequencing methods, the fragments involve errors and gaps. It should be noted that the former derived from the wrong determination of allele’s measure; while, the latter is related to the low-confidence measures of allele positions. SIH attempts to assign each fragment to the right chromosome copy. Then, it detects and corrects the errors to reconstruct the desired haplotypes. In order to solve this problem, several models have been proposed which minimum SNP removal (MSR) [14], minimum fragment removal (MFR) [14], and minimum error correction (MEC) [15] are the chief models. Among the existing models, MEC is more efficient and has been applied in several approaches [16–19]. The aim of this model is to find and correct the errors by applying the minimum letter changes in the input fragments. It has been proved that all of the models are NP-Hard [14]. Most of the current methods construct a weighted graph such that each fragment corresponds with a vertex and the weight of each edge represents the amount of similarity between the connecting fragments. Based on the used model, each method transforms the built graph into a bipartite graph. For example in the MEC model, this is performed by deleting the least number of conflicting edges. AROHap [19] and FCMHap [20] are two recent methods which have been addressed the problem according to the MEC model. The first, through the use of asexual reproduction optimization (ARO) algorithm, attempts to improve the fitness function which is designed based on the MEC model. The second, by exploiting the Fuzzy c-means (FCM) clustering algorithm tries to improve the initial haplotypes iteratively. It is worthwhile noting that the method divides the input fragments into two groups and the haplotypes are obtained as the center of the clusters. However, some popular methods such as MCMC [21] and HapCUT [16] build the graph in a different way. These methods start with a set of arbitrary sequences as initial haplotypes and improve it step by step regarding the input fragments. They make a similar weighted graph in their distinctive model; but instead of fragments, SNPs are the vertices. Each pair of SNPs is connected if they are covered by at least one input fragments. The weight of each edge describes the amount of consistency with their corresponding positions in the current haplotypes. Albeit, this model efficiently describes the consistency of the current haplotype with the input fragments; but the existence of gaps and noise may lead to achieving inaccurate weights [22].

In this paper, we propose a fast and accurate method to solve haplotype reconstruction named NCMHap which involves two steps. First, a weighted fuzzy conflict graph is made such that each node corresponds with an input fragment and the weight of each edge represents the measurement of incompatibility between the corresponding input fragments. By removing the least of conflicting edges based on the MEC model and bi-partitioning the input fragments, an initial fragment clustering is obtained. Next, to decrease the effect of noise and outliers on the obtained clusters, the Neutrosophic c-means (NCM) clustering method is applied. NCM by assigning a coefficient to each input fragment can reduce the noise effects on the clustering process. The performance of the proposed method is validated with both simulated and real datasets. According to the obtained results, by selecting appropriate measures for the parameters of NCM, our method can provide high throughput reconstructed haplotypes close to the optimal.

## Results

In this section, the performance of NCMHap is evaluated by using two simulated and publicly available real datasets.

### Setting the parameters

The proposed method was implemented in MATLAB and all experiments were completed on a Core i5 Intel with 2.7 GHz and 8G RAM. Among the parameters, m and $\varepsilon$ are common with fuzzy c-means clustering which usually are set by 2 and $10^{ - 5}$, respectively. The other parameters i.e. $\delta$, $w_{1}$,$w_{2}$, and $w_{3}$ are set as $25$, 0.7, 0.2, and 0.1, respectively, which were tuned by trial and error. For this aim, similar to the study of Guo and Sengur [23], a grid search of the trade-off constant $\delta$ on {5, 10, 15,…, 30} and $w_{1}$,$w_{2}$, and $w_{3}$ on {0.1, 0.2, 0.3,…, 0.9} was performed to find the optimal results. Similar to the previous works [16, 19, 22, 24–27], Reconstruction rate (RR) measure is used to evaluate the quality of the obtained haplotypes.

### Competitor methods

In this experiment, NCMhap is compared with a set of state-of-the-art and well-known methods. Some important notes about these competitors are described as follows:

1. H-PoP [26] clusters the DNA reads into k groups such that the elements of each cluster have minimum distance with each other while are far from the reads of the other clusters. Moreover, it exploits the genotype information to improve the reconstructed haplotypes.
2. SCGD [28] is a heuristic-based method that models SIH as the low-rank matrix factorization problem and represents a modified of the gradient descent algorithm to solve the problem.
3. FastHap [25] is an iterative based method which models the similarities between the input fragments with a weighed fuzzy conflict graph.
4. FCMHap [20] uses the Fuzzy C-means clustering method to divide the input fragments into two segments with minimum MEC measure.
5. HGHap [22] exploits the hypergraph model to describe the similarities between the input fragments more precisely.
6. AROHap [19] is a nature-inspired method that utilizes the Asexual Reproduction optimization method to cluster the input fragments with the best MEC score.
7. ALTHap [27] is an iterative algorithm that formulates the haplotype assembly problem as a sparse tensor decomposition.
8. HRCH [29] utilizes a chaotic viewpoint to reconstruct haplotypes. For this aim, the obtained haplotypes are mapped to some coordinate series by applying chaos game representation. Then, the positions with low confidences are improved by using a local projection.

## Simulated data

In order to evaluate the performance of the proposed method, first, the experiments have been carried out on a widely used dataset named as Geraci’s dataset [30]. It was provided by the international Hapmap project which is based on 22 chromosomes of 269 different individuals.

The individuals have been nominated from Japan (JPT), China (HCB), Nigeria (YR), and Utah (CEU). Haplotype length (*l*), coverage (c), and error rate (e) are the main parameters which $l = \left\{ {100, 350, 700} \right\}$, $c = \left\{ {3, 5, 8, 10} \right\}$ and $e = \left\{ {0.1, 0.2, 0.3} \right\}$. It should be noted that for each combination of these parameters there are 100 instances.

Since the proposed method involves two steps, it can be desired to evaluate the influence of each step independently. For this purpose, the initial clustering, NCM algorithm, and NCMHap are separately executed on the Geraci’s dataset. The obtained results for haplotypes with length 100, 350, and 700 are listed in Tables 1, 2 and 3 respectively. It should be noted that the first two columns in these tables are the error rate e and the coverage c, respectively. In each table, The NCM column represents the results when it starts with a random initial guess for each cluster center.

**Table 1:** The average reconstruction rate over 100 instances with length 100

e	c	Initial	NCM	NCMhap
0.1	3	0.657	0.817	0.916
	5	0.677	0.846	0.971
	8	0.676	0.946	0.983
	10	0.675	0.885	0.989
0.2	3	0.611	0.693	0.822
	5	0.620	0.730	0.907
	8	0.616	0.793	0.931
	10	0.633	0.826	0.936
0.3	3	0.554	0.581	0.684
	5	0.568	0.677	0.759
	8	0.565	0.653	0.816
	10	0.564	0.692	0.843

**Table 2:** The average reconstruction rate over 100 instances with length 350

e	c	Initial	NCM	NCMhap
0.1	3	0.639	0.688	0.953
	5	0.655	0.718	0.982
	8	0.664	0.805	0.989
	10	0.665	0.812	0.993
0.2	3	0.586	0.632	0.856
	5	0.600	0.678	0.921
	8	0.610	0.720	0.939
	10	0.619	0.703	0.948
0.3	3	0.527	0.580	0.712
	5	0.539	0.583	0.803
	8	0.540	0.590	0.850
	10	0.547	0.611	0.870

**Table 3:** The average reconstruction rate over 100 instances with length 700

e	c	Initial	NCM	NCMhap
0.1	3	0.635	0.704	0.958
	5	0.656	0.761	0.984
	8	0.647	0.745	0.990
	10	0.657	0.746	0.994
0.2	3	0.584	0.634	0.865
	5	0.607	0.624	0.925
	8	0.598	0.714	0.938
	10	0.599	0.708	0.946
0.3	3	0.520	0.545	0.720
	5	0.538	0.569	0.808
	8	0.535	0.590	0.849
	10	0.545	0.618	0.958

It can be seen in the last column of Tables 1, 2 and 3, the synergistic of these steps achieved promising results which completely outperform the other cases.

**Fig. 1:**
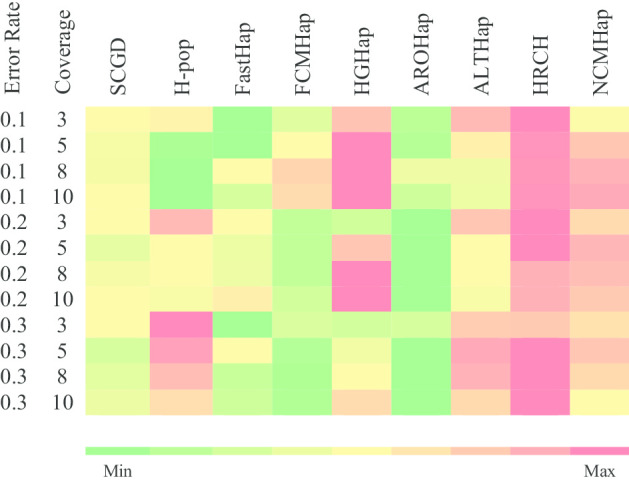
Performance comparison of NCMHap and other methods on the Geraci's dataset [30] with haplotype block length *l* = 100

**Fig. 2:**
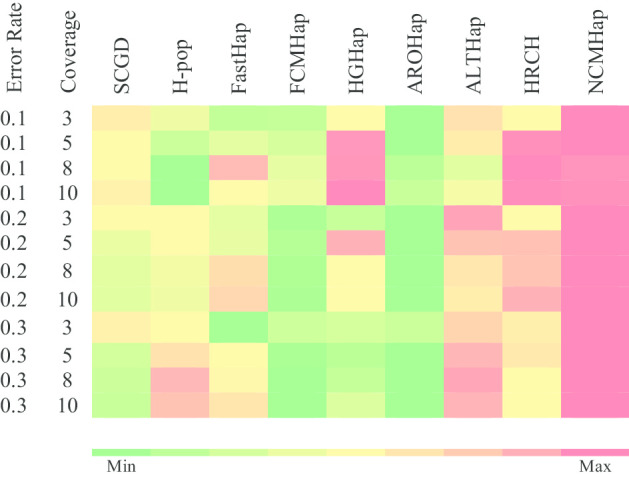
Performance comparison of NCMHap and other methods on the Geraci's dataset [30] with haplotype block length *l* = 350

**Fig. 3:**
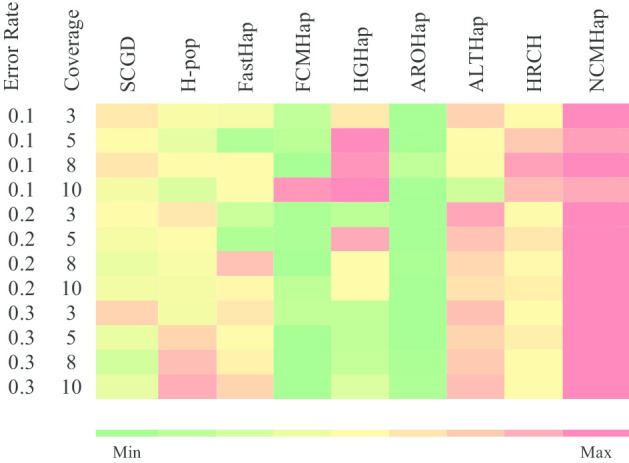
Performance comparison of NCMHap and other methods on the Geraci's dataset [30] with haplotype block length *l* = 700

Figures 1, 2 and 3 demonstrate the comparison of RRs obtained from the run of the NCMHap as well as the benchmarking algorithms on Geraci’s dataset for haplotypes with length 100, 350, and 700 respectively. Each figure represents a heatmap. The color of each row ranges from green i.e. the minimum RR to red i.e. the maximum RR. It should be noted that each heatmap cell is obtained based on computing the average over 100 data samples.

By investigating the heatmap of Fig. 1, it reveals that the proposed method can provide high-quality results and completely comparable against the other approaches. Comparing the results demonstrates that the proposed method completely outperforms SCGD, FastHap, FCMHap, and AROHap algorithms in all parameters.

As can be seen in Fig. 2, by increasing the length of fragments, the quality of the obtained haplotypes is efficiently improved. Particularly, when the amount of noise is increased, it can preserve the quality of reconstructed haplotypes against the other approaches and in most cases outperforms the benchmarking methods.

Finally, as demonstrated in Fig. 3, for input fragments with length 700, except for one situation, NCMHap has achieved better reconstruction rates than any other algorithms. It should be noted that the obtained RR measures are listed in Additional file 1: Tables S1–S3.

Investigating the obtained results demonstrates that the proposed method can provide high performance in dealing with long input fragments. In fact, increasing the length of input fragments as well as the rate of coverage enable the proposed method to compute the similarity between the fragments more precisely. Moreover, increasing the length of input fragments can aid to identify and decrease the effect of outliers more accurately.

**Fig. 4:**
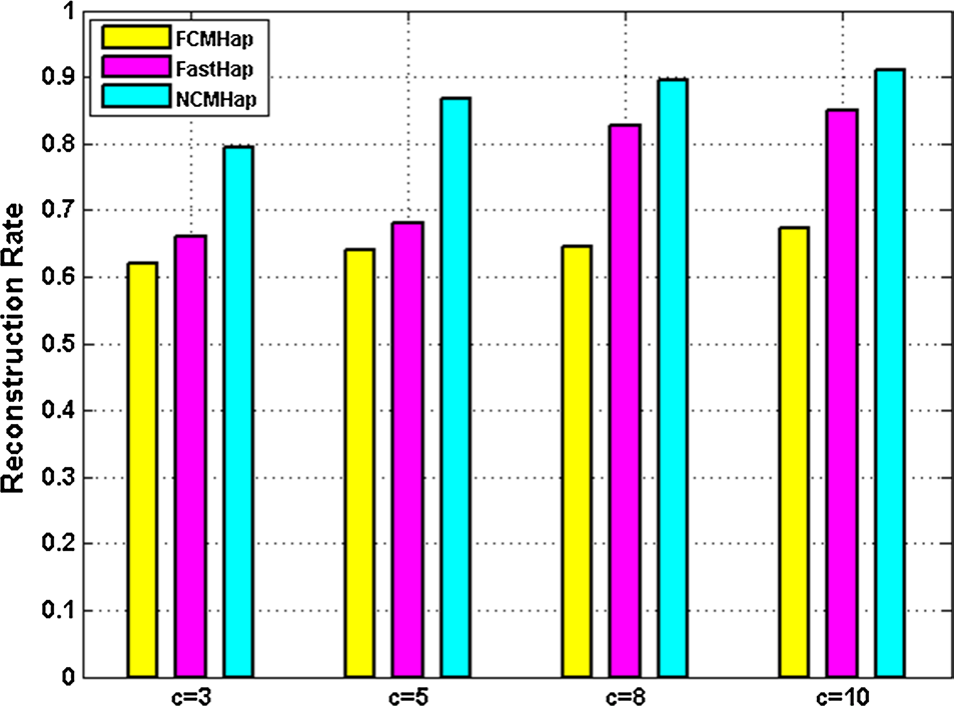
Comparison the reconstruction rate of the proposed method against FastHap and FCMHap methods while $e \ge 0.2$

Since the Neutrosophic c-means clustering is a developed form of Fuzzy c-means method and moreover NCMHap like Fast method uses weighted fuzzy conflict graph to model the similarity between the input fragments, its performance is compared against FCMHap and FastHap approaches when it deals with long block haplotypes and a huge amount of noise. Figure 4 demonstrates the quality of obtained results for haplotypes with length $700$ and error rate $e \ge 0.2$.

It is apparent the results of the proposed method are valuable against comparing methods in dealing with input fragments with a high error rate.

## Experimental data

**Fig. 5:**
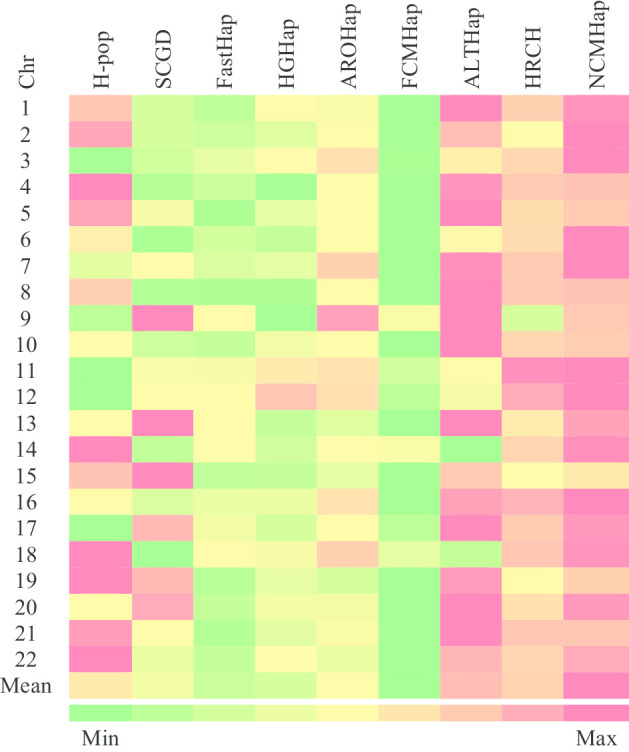
The reconstruction rate for the proposed method, H-pop, SCGD, FastHap, HGHap, AROHap, FCMHap, ALTHap, and HRCH applied to the experimental dataset NA12878 dataset provided by the 1000 genome project

For more investigation, we tested the performance of our method on a real dataset which involves data provided by the 1000 genome project [31]. This data belongs to an individual NA12878 [32] which is frequently used to investigate the performance of the existing SIH methods. Moreover, the trio-phased variant calls from the GATK resource bundle [33] was used as the true haplotypes. The represented heatmap in Fig. 5, illustrates the reconstruction rate of the proposed method as well as H-PoP [26], SCGD [28], FastHap [25], HGHap [22], AROHap [19], ALTHap [27], and HRCH [29]. The obtained results demonstrate that our method achieves the highest and second-highest RRs for most of the chromosomes.

Evaluating the obtained results on both simulated and experimental datasets demonstrates that the proposed method can provide promising reconstructed haplotypes in dealing with low-quality sequencing data. Moreover, in the worst case, NCMHap can solve the problem in less than 3 min which this runtime is suitable against the existing approaches. It should be noted that the running times of the competitor methods are represented in Additional file 1: Tables S5–S8.

## Discussion

Haplotypes could have profound impacts on personalized medicine. Moreover, it can be used for the study of human evolutionary history. Haplotype assembly includes assembling a pair of haplotypes from a huge amount of individual's aligned DNA sequence fragments. Nevertheless, the quality of the reconstructed haplotypes is poor due to the sparsity as well as the amount of noise in the sequenced fragments. NCMHap reconstructs the haplotypes based on the Neutrosophic c-means (NCM) clustering algorithm.

By evaluating the results of NCMHap on both simulated and real datasets, we found that the proposed approach could effectively overcome the challenge of the occurrence of noise in the input fragments, and could provide promising results compared with current methods.

In order to increase the convergence speed of NCM as well as improving the accuracy of the results, as a pre-processing step, a weighted fuzzy conflict graph is constructed, where the nodes correspond with the fragments and each edge represents the similarity of the corresponding fragments. By partitioning the graph, and clustering the input fragments, an initial haplotype is obtained which feds to the next step.

According to the obtained results, it can be concluded that NCMHap provides comparable performance while offering reasonable execution speed. Moreover, when the length of input fragments is increased, it can outperform other methods in terms of the reconstruction rate. By utilizing NCM, the proposed method can more accurately identify long noisy input fragments as outliers and decreases their effects on the reconstructing of haplotypes.

It should be noted that the performance of the proposed method relied on initializing the parameters of NCM. Consequently, these parameters should be tuned appropriately.

Moreover, although NCMHap performance is already good enough compared with other existing methods, it can only be applied for diploid organisms. Therefore, further research should be conducted to reconstruct haplotypes for the polyploid organisms.

## Conclusion

In this paper, we presented a method based on the Neutrosophic c-means (NCM) clustering algorithm for haplotype assembly problem. Time complexity and handling high error rate datasets are the main challenges of the existing methods. Due to improving the NCM’s convergence speed, the proposed method consists of two phases. First, the input fragments are divided into two partitions based on their similarities. Second, information of bi-partitioning is employed as initial centers by the NCM clustering method. Applying the information in NCM can improve the speed of convergence and decrease the number of iterations. Using simulated and real datasets, the proposed method provides promising performance, in terms of reconstruction rate and running time, to the current methods. Moreover, the obtained results demonstrate that the proposed method provides high efficiency to reconstruct haplotypes with a high-error-rate.

As demonstrated in a series of recent publications (see, e.g., [22, 34–37]) in developing new prediction methods, user friendly and publicly accessible web-servers will significantly enhance their impacts [26], we shall make efforts in our future work to provide a web-server for the prediction method presented in this paper. Also, the source code of NCMHap is freely available at https://github.com/Fatemeh-Zamani/NCMHap.

## Methods

### Problem formulation

**Fig. 6:**
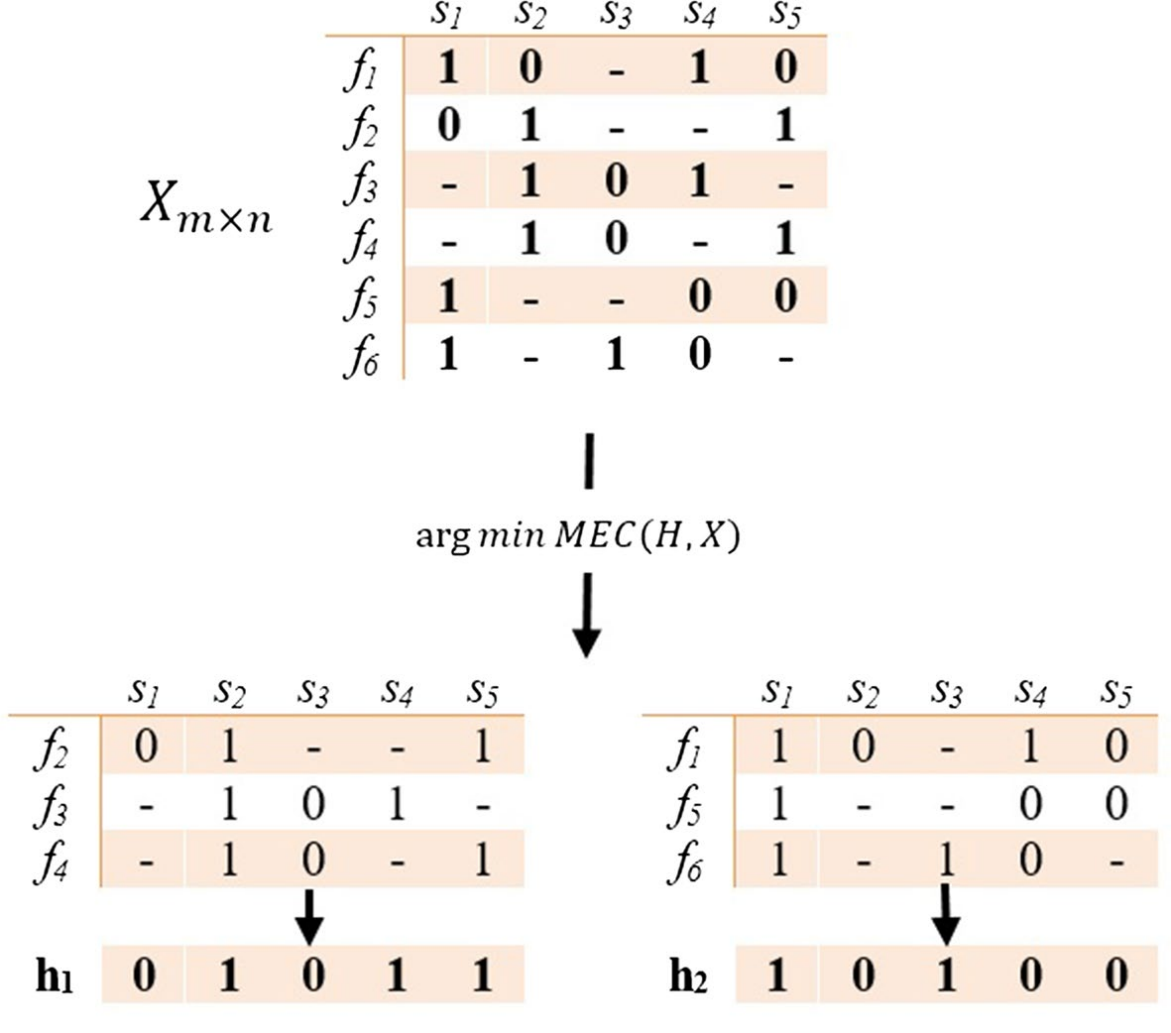
An example of haplotype reconstruction using the MEC model [39]

As can be seen in Fig. 6, $X_{m \times n}$ is a SNP matrix where each row corresponds with an input fragment with length *n*. Since in most cases, there are two alleles at each SNP site, for simplicity, the major and minor alleles are represented by 0 and 1 respectively. It should be noted that if a SNP value cannot be determined with enough confidence, it is indicated by ‘−’.

Let $f_{i}$ and $f_{j}$ are two arbitrary input fragments. The Hamming distance (*HD*) can describe their similarity as below:

1 $$HD\left( {f_{i}, f_{j} } \right) = \mathop \sum \limits_{k = 1}^{n} D\left( {f_{ik}, f_{jk} } \right)$$

2 $$D\left( {a,b} \right) = \left\{ {\begin{array}{*{20}l} 1 \hfill & {\quad if\;a, b \ne^{\prime} -^{\prime}\;and\;a \ne b} \hfill \\ 0 \hfill & {\quad else} \hfill \\ \end{array} } \right.$$

where $f_{i}$ and $f_{j}$ are compatible if $HD = 0$, else they are in conflict. In other words, when $HD\left( {f_{i}, f_{j} } \right)$ equals zero, it can be concluded that these fragments are originated from the same chromosome copy, otherwise, the fragments belong to different chromosome copy, or some of their positions are destroyed by noise. To solve the problem, the fragments of the SNP matrix must be divided into two clusters such that the elements of each cluster will be compatible by the minimum number of letter flips i.e. MEC measure is minimized. Then, the center of each cluster equals with its corresponding haplotype. Figure 6, demonstrates the haplotype reconstruction in the diploid genome, *X* is SNP matrix which divided into two parts and $H = \{ h_{1}, h_{2} \}$ involves the reconstructed haplotypes of each cluster**.**

In order to evaluate the quality of the obtained haplotypes, reconstruction rate (RR) [38] and MEC score are two useful measurements. Let $\hat{H}$ and $H$ contain the reconstructed haplotypes and the original haplotypes respectively. The RR describes the similarity between $\hat{H}$ and $H$ that it is computed as below.

3 $$RR_{{\left( {\hat{H}.H} \right)}} = 1 - \frac{{min\left( {HD\left( {\hat{h}_{1} ,h_{1} } \right) + HD\left( {\hat{h}_{2} ,h_{2} } \right),HD\left( {\hat{h}_{1} ,h_{2} } \right) + HD\left( {\hat{h}_{2} ,h_{1} } \right)} \right)}}{2n}$$

### Neutrosophic c-means (NCM) algorithm

As stated previously, fragment clustering is an important phase of the haplotype assembly. Also, a huge amount of noise and gaps in the input fragments have made this phase as a challenging task. In order to perform this phase efficiently, we consider the Neutrosophic c-means (NCM) clustering algorithm. The algorithm computes the degrees belonging to the determinant and indeterminate clusters at the same time for each of the data points [23, 40]. Outlier and noise data are considered as Indeterminate clusters. Therefore, the NCM algorithm can detect outliers and noisy data. Also, by using some relevant functions, it can decrease the undesirable effects of noise and outliers on the clustering process. For this purpose, the NCM algorithm minimizes the objective function given in Eq. (4) through an iterative process, whereby the centers of the clusters are determined with the least error and the clustering accuracy is improved.

4 $$J\left( {T,I,F,C} \right) = \mathop \sum \limits_{i = 1}^{N} \mathop \sum \limits_{j = 1}^{C} \left( {w_{1} T_{ij} } \right)^{m} \left\| {x_{i} - c_{j} } \right\|^{2} + \mathop \sum \limits_{i = 1}^{N} \left( {w_{2} I_{i} } \right)^{m} \left\| {x_{i} - \overline{c}_{{i{ }max}} } \right\|^{2} + \mathop \sum \limits_{i = 1}^{N} \delta^{2} \left( {w_{3} F_{i} } \right)^{m}$$

5 $$\overline{c}_{{i{ }max}} = \frac{{c_{{p_{i} }} + c_{{q_{i} }} }}{2}$$

6 $$p_{i} = \mathop {\text{arg max}}\limits_{j = 1,2, \ldots ,C} \left( {T_{ij} } \right)$$

7 $$q_{i} = \mathop {\text{arg max}}\limits_{{j \ne p_{i} \cap j = 1,2, \ldots ,C}} \left( {T_{ij} } \right)$$

In the above relations, $T_{{{\text{ij}}}}$ is defined as the degree to determinant clusters, $I_{{\text{i}}}$ is the degree to the boundary clusters, $F_{{\text{i}}}$ is the degree belonging to the outlier data set, *N* number of data points, *C* number of clusters, *w* weighting factor, *m* is a fuzzification constant, *x*_*i*_ is a data point, and *δ* is the number of objects that are considered as outliers. $\overline{c}_{i max}$ is a constant that is calculated for each data point according to Eq. (5). This parameter is used to precisely determine the value of function $I_{{\text{i}}}$, because the degree of indeterminacy of each data point depends on the two largest definite clusters close to it, namely Eqs. (6) and (7). The cluster centers $c_{j}$ and membership $T_{{{\text{ij}}}}$, $I_{{\text{i}}}$, and $F_{{\text{i}}}$ are updated by Eqs. (8–11) respectively, where k is the iteration step.

8 $$c_{j} = \frac{{\mathop \sum \nolimits_{i = 1}^{N} \left( {w_{1} T_{ij} } \right)^{m} x_{i} }}{{\mathop \sum \nolimits_{i = 1}^{N} \left( {w_{1} T_{ij} } \right)^{m} }}$$

9 $$T_{ij} = \frac{K}{{w_{1} }}\left( {x_{i} - c_{j} } \right)^{{ - \left( {{\raise0.7ex\hbox{$2$} \!\mathord{\left/ {\vphantom {2 {m - 1}}}\right.\kern-\nulldelimiterspace} \!\lower0.7ex\hbox{${m - 1}$}}} \right)}}$$

10 $$I_{i} = \frac{K}{{w_{2} }}\left( {x_{i} - \overline{c}_{{i{ }max}} } \right)^{{ - \left( {{\raise0.7ex\hbox{$2$} \!\mathord{\left/ {\vphantom {2 {m - 1}}}\right.\kern-\nulldelimiterspace} \!\lower0.7ex\hbox{${m - 1}$}}} \right)}}$$

11 $$F_{i} = \frac{K}{{w_{3} }}\delta^{{ - \left( {{\raise0.7ex\hbox{$2$} \!\mathord{\left/ {\vphantom {2 {m - 1}}}\right.\kern-\nulldelimiterspace} \!\lower0.7ex\hbox{${m - 1}$}}} \right)}}$$

### NCMHap method

**Fig. 7:**
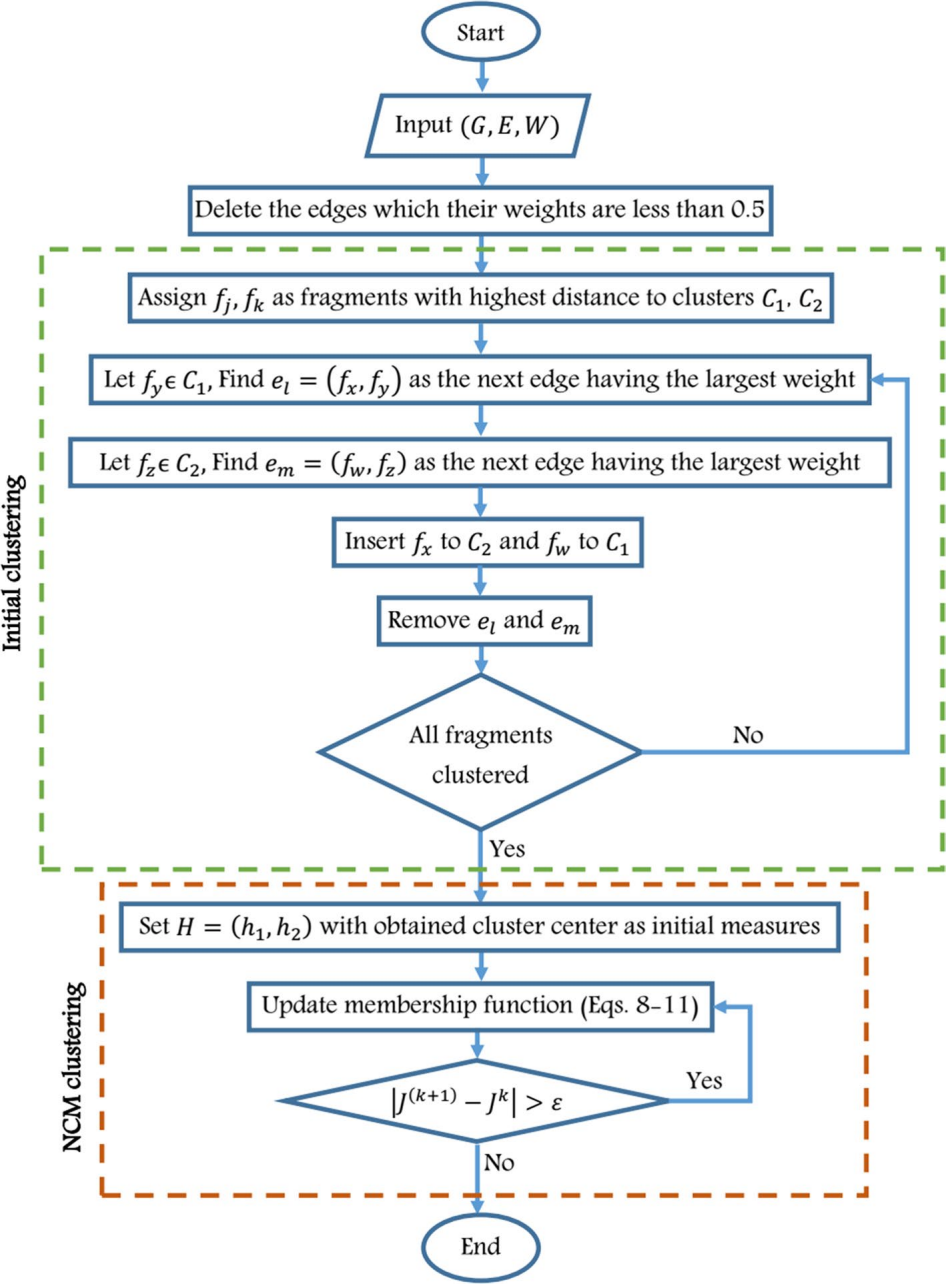
Flowchart of the proposed method

As can be seen in Fig. 7, the proposed method involves two main steps. First, in order to provide an initial clustering of the input fragments, a weighted graph, called fuzzy conflict graph, is constructed based on the SNP matrix. In this graph, fragments are considered as vertices, and the weight of each edge is the normalized Hamming distance (*NHD*) between corresponding fragments. This measure is given as follows:

12 $$NHD\left( {f_{i} ,f_{j} } \right) = \frac{1}{{S_{ij} }}\mathop \sum \limits_{k = 1}^{n} D\left( {f_{ik} ,f_{jk} } \right)$$

In the above relations, *f*_*i*_ and *f*_*j*_ are two fragments of *X*, *S*_*ij*_ denotes the number of columns (SNPs) that are covered by either *f*_*ik*_ or *f*_*jk*_ in *X*. In fact, *S*_*ij*_ is a normalization factor that allows us to normalize the distance between the two fragments such that the resulting distance ranges from 0 to 1, and *n* represents the number of SNPs.

After constructing the graph, the edges with weight of 0.5 are removed because they do not provide sufficient information about the clustering of the connected fragments.

Next, an edge with the highest weight is found from the obtained graph and its connecting nodes (fragments) are assigned to different clusters (i.e. *C*_1_ and *C*_2_). Then, in an iterative manner, for each cluster (*C*_*i*_*, i* = 1,2), a node with highest distance from the cluster is found. Then, it is assigned to the opposite cluster. This step is repeated until all nodes will be assigned to the clusters.

In the second phase, the initial clustering is given to the NCM algorithm. The centers of each cluster are considered as the primary centers in the NCM algorithm. Initial clustering can improve the convergence speed of the NCM algorithm. This algorithm determines the impact of fragments on clustering based on the three membership functions introduced and is able to reduce the impact of noise or outliers on the clustering process and consequently, the accuracy of clustering will be increased. Therefore, clustering is achieved by repeating the optimal objective function and the membership degree of the determinant and indeterminate clusters and the centers of the clusters in each iteration will be updated by Eqs. (8–11). The iteration is repeated until the difference between cluster centers at two successive iterations is greater than $\varepsilon$. Finally, the center of obtained clusters construct the set of reconstructed haplotypes.

## Supplementary information

**Additional file 1:** **Table S1.** Performance comparison of NCMHap and other methods on the Geraci's dataset with haplotype block length *l = 100*. Each element in this table is the average value of each 100 data samples. **Table S2.** Performance comparison of NCMHap and other methods on the Geraci's dataset with haplotype block length *l = 350*. Each element in this table is the average value of each 100 data samples. **Table S3.** Performance comparison of NCMHap and other methods on the Geraci's dataset with haplotype block length *l = 700*. Each element in this table is the average value of each 100 data samples. **Table S4.** The reconstruction rate for the proposed method, H-pop, SCGD, FastHap, HGHap, AROHap, FCMHap, ALTHap, and HRCH applied to the experimental dataset NA12878 dataset provided by 1000 genome project. **Table S5.** The average of running time of NCMHap and other methods on the Geraci's dataset with haplotype block length *l = 100* (In seconds). **Table S6.** The average of running time of NCMHap and other methods on the Geraci's dataset with haplotype block length *l = 350* (In seconds). **Table S7.** The average of running time of NCMHap and other methods on the Geraci's dataset with haplotype block length *l = 700* (In seconds). **Table S8.** The average of running time for the proposed method, H-pop, SCGD, FastHap, HGHap, AROHap, FCMHap, ALTHap, and HRCH applied to the experimental dataset NA12878 dataset provided by 1000 genome project (In seconds).

## Data Availability

The datasets generated and analyzed during the current study are available from the corresponding author on reasonable request. Moreover, the source code is available in: https://github.com/Fatemeh-Zamani/NCMHap.

## Abbreviations

SIH: Single individual haplotype
NCM: Neutrosophic c-means
NGS: Next generation sequencing
SNP: Single nucleotide polymorphism
MSR: Minimum SNP removal
MFR: Minimum fragment removal
MEC: Minimum error correction
ARO: Asexual reproduction optimization
FCM: Fuzzy c-means
RR: Reconstruction rate
NHD: Normalized Hamming distance
