# Supplementary material for: NCMHap: a novel method for haplotype reconstruction based on Neutrosophic c-means clustering
Source: BMC Bioinformatics. 2020 Oct 22;21:475. doi: 10.1186/s12859-020-03775-0 (PMC7579908; doi:10.1186/s12859-020-03775-0)
Supplement: Supplementary file 1 — Additional file 1: Table S1. Performance comparison of NCMHap and other methods on the Geraci's dataset with haplotype block length l = 100. Each element in this table is the average value of each 100 data samples. Table S2. Performance comparison of NCMHap and other methods on the Geraci's dataset with haplotype block length l = 350. Each element in this table is the average value of each 100 data samples. Table S3. Performance comparison of NCMHap and other methods on the Geraci's dataset with haplotype block length l = 700. Each element in this table is the average value of each 100 data samples. Table S4. The reconstruction rate for the proposed method, H-pop, SCGD, FastHap, HGHap, AROHap, FCMHap, ALTHap, and HRCH applied to the experimental dataset NA12878 dataset provided by 1000 genome project. Table S5. The average of running time of NCMHap and other methods on the Geraci's dataset with haplotype block length l = 100 (In seconds). Table S6. The average of running time of NCMHap and other methods on the Geraci's dataset with haplotype block length l = 350 (In seconds). Table S7. The average of running time of NCMHap and other methods on the Geraci's dataset with haplotype block length l = 700 (In seconds). Table S8. The average of running time for the proposed method, H-pop, SCGD, FastHap, HGHap, AROHap, FCMHap, ALTHap, and HRCH applied to the experimental dataset NA12878 dataset provided by 1000 genome project (In seconds). [file 12859_2020_3775_MOESM1_ESM.docx]

Table S1. Performance comparison of NCMHap and other methods on the Geraci's dataset with haplotype block length *l* *= 100*. Each element in this table is the average value of each 100 data samples.

| Error | Coverage | SCGD | H-pop | FastHap | FCMHap | HGHap | AROHap | ALTHap | HRCH | NCMHap |
| --- | --- | --- | --- | --- | --- | --- | --- | --- | --- | --- |
| 0.1 | 3 | 0.918 | 0.921 | 0.823 | 0.882 | 0.941 | 0.844 | 0.944 | 0.957 | 0.916 |
|  | 5 | 0.944 | 0.919 | 0.917 | 0.948 | 0.989 | 0.922 | 0.953 | 0.987 | 0.971 |
|  | 8 | 0.948 | 0.900 | 0.955 | 0.971 | 0.994 | 0.945 | 0.945 | 0.991 | 0.983 |
|  | 10 | 0.959 | 0.892 | 0.926 | 0.972 | 0.997 | 0.92 | 0.943 | 0.995 | 0.989 |
| 0.2 | 3 | 0.806 | 0.836 | 0.806 | 0.739 | 0.752 | 0.711 | 0.831 | 0.851 | 0.822 |
|  | 5 | 0.825 | 0.865 | 0.834 | 0.772 | 0.899 | 0.736 | 0.865 | 0.926 | 0.907 |
|  | 8 | 0.861 | 0.873 | 0.849 | 0.793 | 0.966 | 0.760 | 0.873 | 0.941 | 0.931 |
|  | 10 | 0.886 | 0.878 | 0.899 | 0.835 | 0.981 | 0.788 | 0.878 | 0.956 | 0.936 |
| 0.3 | 3 | 0.671 | 0.717 | 0.578 | 0.629 | 0.621 | 0.627 | 0.694 | 0.695 | 0.684 |
|  | 5 | 0.676 | 0.784 | 0.711 | 0.648 | 0.698 | 0.638 | 0.780 | 0.798 | 0.759 |
|  | 8 | 0.740 | 0.835 | 0.700 | 0.664 | 0.79 | 0.649 | 0.841 | 0.861 | 0.816 |
|  | 10 | 0.798 | 0.855 | 0.732 | 0.675 | 0.856 | 0.653 | 0.857 | 0.881 | 0.843 |

Table S2. Performance comparison of NCMHap and other methods on the Geraci's dataset with haplotype block length *l* *= 350*. Each element in this table is the average value of each 100 data samples.

| Error | Coverage | SCGD | H-pop | FastHap | FCMHap | HGHap | AROHap | ALTHap | HRCH | NCMHap |
| --- | --- | --- | --- | --- | --- | --- | --- | --- | --- | --- |
| 0.1 | 3 | 0.941 | 0.921 | 0.872 | 0.873 | 0.939 | 0.844 | 0.943 | 0.939 | 0.953 |
|  | 5 | 0.945 | 0.912 | 0.927 | 0.919 | 0.979 | 0.892 | 0.951 | 0.981 | 0.982 |
|  | 8 | 0.950 | 0.896 | 0.977 | 0.934 | 0.988 | 0.908 | 0.930 | 0.991 | 0.989 |
|  | 10 | 0.952 | 0.889 | 0.947 | 0.935 | 0.995 | 0.910 | 0.941 | 0.994 | 0.993 |
| 0.2 | 3 | 0.813 | 0.813 | 0.763 | 0.671 | 0.712 | 0.659 | 0.849 | 0.813 | 0.856 |
|  | 5 | 0.817 | 0.860 | 0.811 | 0.719 | 0.905 | 0.691 | 0.896 | 0.897 | 0.921 |
|  | 8 | 0.832 | 0.871 | 0.912 | 0.728 | 0.899 | 0.709 | 0.908 | 0.922 | 0.939 |
|  | 10 | 0.838 | 0.873 | 0.923 | 0.733 | 0.907 | 0.719 | 0.913 | 0.937 | 0.948 |
| 0.3 | 3 | 0.637 | 0.629 | 0.575 | 0.597 | 0.602 | 0.595 | 0.664 | 0.640 | 0.712 |
|  | 5 | 0.661 | 0.744 | 0.720 | 0.614 | 0.632 | 0.609 | 0.777 | 0.737 | 0.803 |
|  | 8 | 0.690 | 0.830 | 0.790 | 0.626 | 0.675 | 0.628 | 0.838 | 0.788 | 0.850 |
|  | 10 | 0.700 | 0.850 | 0.833 | 0.631 | 0.742 | 0.635 | 0.856 | 0.821 | 0.870 |

Table S3. Performance comparison of NCMHap and other methods on the Geraci's dataset with haplotype block length *l* *= 700.* Each element in this table is the average value of each 100 data samples.

| Error | Coverage | SCGD | H-pop | FastHap | FCMHap | HGHap | AROHap | ALTHap | HRCH | NCMHap |
| --- | --- | --- | --- | --- | --- | --- | --- | --- | --- | --- |
| 0.1 | 3 | 0.934 | 0.919 | 0.917 | 0.834 | 0.934 | 0.801 | 0.941 | 0.928 | 0.958 |
|  | 5 | 0.951 | 0.923 | 0.872 | 0.881 | 0.990 | 0.862 | 0.951 | 0.972 | 0.984 |
|  | 8 | 0.956 | 0.945 | 0.945 | 0.883 | 0.987 | 0.899 | 0.943 | 0.983 | 0.990 |
|  | 10 | 0.973 | 0.951 | 0.983 | 0.996 | 0.997 | 0.912 | 0.942 | 0.992 | 0.994 |
| 0.2 | 3 | 0.796 | 0.811 | 0.703 | 0.652 | 0.677 | 0.644 | 0.852 | 0.797 | 0.865 |
|  | 5 | 0.829 | 0.854 | 0.681 | 0.672 | 0.910 | 0.662 | 0.896 | 0.869 | 0.925 |
|  | 8 | 0.832 | 0.868 | 0.916 | 0.686 | 0.884 | 0.695 | 0.905 | 0.885 | 0.938 |
|  | 10 | 0.860 | 0.869 | 0.896 | 0.746 | 0.894 | 0.698 | 0.909 | 0.900 | 0.946 |
| 0.3 | 3 | 0.652 | 0.600 | 0.627 | 0.592 | 0.592 | 0.588 | 0.674 | 0.602 | 0.720 |
|  | 5 | 0.659 | 0.733 | 0.682 | 0.599 | 0.621 | 0.598 | 0.735 | 0.699 | 0.808 |
|  | 8 | 0.662 | 0.804 | 0.741 | 0.606 | 0.646 | 0.613 | 0.793 | 0.729 | 0.849 |
|  | 10 | 0.714 | 0.844 | 0.805 | 0.606 | 0.696 | 0.618 | 0.829 | 0.759 | 0.870 |

Table S4. The reconstruction rate for the proposed method, H-pop, SCGD, FastHap, HGHap, AROHap, FCMHap, ALTHap, and HRCH applied to the experimental dataset NA12878 dataset provided by 1000 genome project.

| Chr | H-pop | SCGD | FastHap | HGHap | AROHap | FCMHap | ALTHap | HRCH | NCMHap |
| --- | --- | --- | --- | --- | --- | --- | --- | --- | --- |
| 1 | 0.957 | 0.925 | 0.919 | 0.937 | 0.935 | 0.913 | 0.974 | 0.954 | 0.972 |
| 2 | 0.956 | 0.926 | 0.922 | 0.929 | 0.943 | 0.908 | 0.953 | 0.943 | 0.959 |
| 3 | 0.912 | 0.919 | 0.923 | 0.928 | 0.940 | 0.913 | 0.933 | 0.944 | 0.969 |
| 4 | 0.970 | 0.927 | 0.933 | 0.923 | 0.949 | 0.923 | 0.969 | 0.960 | 0.961 |
| 5 | 0.966 | 0.939 | 0.914 | 0.932 | 0.942 | 0.912 | 0.972 | 0.952 | 0.957 |
| 6 | 0.952 | 0.930 | 0.938 | 0.935 | 0.948 | 0.929 | 0.949 | 0.958 | 0.977 |
| 7 | 0.924 | 0.935 | 0.921 | 0.925 | 0.951 | 0.904 | 0.970 | 0.954 | 0.971 |
| 8 | 0.947 | 0.907 | 0.906 | 0.906 | 0.934 | 0.903 | 0.962 | 0.949 | 0.950 |
| 9 | 0.910 | 0.971 | 0.940 | 0.901 | 0.966 | 0.937 | 0.971 | 0.921 | 0.956 |
| 10 | 0.945 | 0.926 | 0.923 | 0.940 | 0.945 | 0.913 | 0.968 | 0.954 | 0.956 |
| 11 | 0.915 | 0.932 | 0.931 | 0.939 | 0.942 | 0.923 | 0.933 | 0.963 | 0.964 |
| 12 | 0.903 | 0.923 | 0.923 | 0.945 | 0.935 | 0.908 | 0.921 | 0.954 | 0.963 |
| 13 | 0.941 | 0.970 | 0.941 | 0.930 | 0.935 | 0.925 | 0.970 | 0.946 | 0.965 |
| 14 | 0.971 | 0.911 | 0.934 | 0.917 | 0.934 | 0.932 | 0.903 | 0.949 | 0.970 |
| 15 | 0.974 | 0.991 | 0.917 | 0.920 | 0.937 | 0.905 | 0.972 | 0.951 | 0.959 |
| 16 | 0.935 | 0.930 | 0.932 | 0.932 | 0.946 | 0.924 | 0.967 | 0.962 | 0.973 |
| 17 | 0.911 | 0.967 | 0.944 | 0.931 | 0.951 | 0.920 | 0.975 | 0.963 | 0.973 |
| 18 | 0.976 | 0.903 | 0.926 | 0.924 | 0.949 | 0.919 | 0.910 | 0.954 | 0.973 |
| 19 | 0.978 | 0.972 | 0.930 | 0.949 | 0.942 | 0.923 | 0.976 | 0.960 | 0.968 |
| 20 | 0.950 | 0.968 | 0.931 | 0.945 | 0.946 | 0.922 | 0.973 | 0.957 | 0.971 |
| 21 | 0.970 | 0.943 | 0.919 | 0.933 | 0.941 | 0.915 | 0.974 | 0.960 | 0.960 |
| 22 | 0.983 | 0.941 | 0.926 | 0.951 | 0.941 | 0.914 | 0.973 | 0.964 | 0.976 |

Table S5. The average of running time of NCMHap and other methods on the Geraci's dataset with haplotype block length *l* *= 100* (In seconds).

| Error | Coverage | SCGD | H-pop | FastHap | FCMHap | HGHap | AROHap | ALTHap | HRCH | NCMHap |
| --- | --- | --- | --- | --- | --- | --- | --- | --- | --- | --- |
| 0.1 | 3 | 0.094 | 0.425 | 0.091 | 0.065 | 0.357 | 1.301 | 0.043 | 7.757 | 0.176 |
|  | 5 | 0.173 | 0.471 | 0.174 | 0.139 | 0.443 | 2.002 | 0.060 | 6.272 | 0.272 |
|  | 8 | 0.773 | 0.541 | 0.440 | 0.334 | 0.682 | 2.324 | 0.088 | 6.161 | 0.613 |
|  | 10 | 2.660 | 0.567 | 0.772 | 0.561 | 0.744 | 3.027 | 0.136 | 5.578 | 0.925 |
| 0.2 | 3 | 0.097 | 0.437 | 0.083 | 0.059 | 0.425 | 1.840 | 0.039 | 7.571 | 0.179 |
|  | 5 | 0.157 | 0.485 | 0.183 | 0.137 | 0.650 | 2.096 | 0.060 | 8.275 | 0.232 |
|  | 8 | 0.537 | 0.527 | 0.518 | 0.307 | 0.834 | 2.017 | 0.094 | 8.525 | 0.510 |
|  | 10 | 3.050 | 0.570 | 0.723 | 0.441 | 1.040 | 2.632 | 0.135 | 6.822 | 0.776 |
| 0.3 | 3 | 0.102 | 0.350 | 0.114 | 0.061 | 0.350 | 1.919 | 0.041 | 9.448 | 0.162 |
|  | 5 | 0.205 | 0.400 | 0.192 | 0.131 | 0.474 | 1.303 | 0.063 | 9.844 | 0.229 |
|  | 8 | 0.643 | 0.454 | 0.461 | 0.283 | 0.720 | 1.930 | 0.093 | 9.462 | 0.493 |
|  | 10 | 3.005 | 0.470 | 0.963 | 0.427 | 0.955 | 2.488 | 0.133 | 10.155 | 0.713 |

Table S6. The average of running time of NCMHap and other methods on the Geraci's dataset with haplotype block length *l* *= 350* (In seconds).

| Error | Coverage | SCGD | H-pop | FastHap | FCMHap | HGHap | AROHap | ALTHap | HRCH | NCMHap |
| --- | --- | --- | --- | --- | --- | --- | --- | --- | --- | --- |
| 0.1 | 3 | 0.403 | 0.707 | 1.444 | 0.772 | 2.263 | 6.013 | 0.210 | 14.62 | 1.517 |
|  | 5 | 1.255 | 0.906 | 4.823 | 1.959 | 5.500 | 8.557 | 0.447 | 13.00 | 3.496 |
|  | 8 | 19.750 | 1.150 | 18.061 | 5.55 | 12.935 | 17.679 | 0.818 | 11.48 | 7.940 |
|  | 10 | 22.836 | 1.300 | 35.922 | 10.10 | 19.192 | 28.498 | 1.962 | 11.27 | 17.16 |
| 0.2 | 3 | 0.417 | 0.762 | 1.494 | 0.792 | 2.226 | 4.948 | 0.220 | 13.37 | 1.610 |
|  | 5 | 1.506 | 0.924 | 4.872 | 1.974 | 5.750 | 8.512 | 0.456 | 14.38 | 3.318 |
|  | 8 | 16.070 | 1.175 | 14.786 | 5.437 | 14.370 | 17.478 | 0.843 | 17.94 | 8.466 |
|  | 10 | 26.652 | 1.331 | 32.024 | 9.212 | 21.356 | 26.242 | 1.843 | 20.37 | 14.11 |
| 0.3 | 3 | 0.432 | 0.916 | 1.255 | 0.813 | 2.559 | 5.161 | 0.226 | 12.48 | 1.607 |
|  | 5 | 1.600 | 0.997 | 2.553 | 2.096 | 6.311 | 8.914 | 0.481 | 14.46 | 3.706 |
|  | 8 | 15.684 | 1.022 | 11.857 | 5.356 | 15.607 | 17.695 | 0.809 | 19.69 | 8.597 |
|  | 10 | 33.916 | 1.330 | 19.952 | 8.621 | 23.727 | 26.004 | 1.948 | 22.53 | 13.78 |

Table S7. The average of running time of NCMHap and other methods on the Geraci's dataset with haplotype block length *l* *= 700* (In seconds).

| Error | Coverage | SCGD | H-pop | FastHap | FCMHap | HGHap | AROHap | ALTHap | HRCH | NCMHap |
| --- | --- | --- | --- | --- | --- | --- | --- | --- | --- | --- |
| 0.1 | 3 | 1.520 | 1.178 | 4.324 | 4.241 | 13.159 | 24.843 | 1.037 | 31.82 | 6.770 |
|  | 5 | 7.319 | 1.886 | 29.161 | 12.28 | 35.123 | 60.621 | 2.062 | 34.37 | 17.55 |
|  | 8 | 61.88 | 3.765 | 141.98 | 41.164 | 85.829 | 164.708 | 4.817 | 43.35 | 72.80 |
|  | 10 | 101.5 | 5.316 | 257.77 | 77.432 | 136.267 | 226.777 | 11.38 | 40.05 | 151.4 |
| 0.2 | 3 | 1.320 | 1.238 | 4.871 | 4.567 | 14.453 | 29.882 | 0.901 | 29.21 | 7.460 |
|  | 5 | 5.744 | 1.979 | 36.862 | 12.368 | 37.972 | 64.612 | 1.938 | 38.73 | 19.36 |
|  | 8 | 64.51 | 3.922 | 110.93 | 37.862 | 100.422 | 157.694 | 4.375 | 55.19 | 65.15 |
|  | 10 | 87.71 | 5.763 | 222.89 | 67.696 | 153.434 | 264.44 | 10.13 | 72.24 | 133.3 |
| 0.3 | 3 | 1.348 | 1.260 | 4.726 | 4.92 | 16.483 | 27.922 | 0.997 | 25.67 | 7.590 |
|  | 5 | 5.216 | 2.082 | 38.79 | 13.785 | 43.084 | 57.402 | 2.056 | 44.44 | 20.83 |
|  | 8 | 79.42 | 4.035 | 127.97 | 39.386 | 117.87 | 128.85 | 4.455 | 70.66 | 71.74 |
|  | 10 | 117.2 | 5.324 | 210.343 | 68.927 | 178.775 | 225.696 | 9.028 | 75.12 | 137.2 |

Table S8. The average of running time for the proposed method, H-pop, SCGD, FastHap, HGHap, AROHap, FCMHap, ALTHap, and HRCH applied to the experimental dataset NA12878 dataset provided by 1000 genome project (In seconds).

| Chr | H-pop | SCGD | FastHap | HGHap | AROHap | FCMHap | ALTHap | HRCH | NCMHap |
| --- | --- | --- | --- | --- | --- | --- | --- | --- | --- |
| 1 | 5.22 | 3.62 | 5.90 | 1.54 | 20.28 | 1.09 | 11.26 | 10.40 | 2.593 |
| 2 | 5.65 | 4.41 | 6.72 | 1.30 | 18.03 | 1.04 | 12.22 | 12.34 | 1.585 |
| 3 | 6.99 | 3.40 | 7.54 | 1.17 | 18.45 | 1.91 | 10.38 | 12.75 | 4.116 |
| 4 | 5.24 | 5.47 | 5.24 | 1.20 | 18.06 | 1.68 | 12.16 | 13.07 | 4.137 |
| 5 | 4.67 | 3.54 | 6.10 | 1.24 | 15.09 | 1.27 | 9.96 | 14.98 | 2.827 |
| 6 | 4.93 | 8.70 | 6.82 | 1.22 | 15.60 | 1.04 | 14.17 | 13.58 | 2.227 |
| 7 | 4.24 | 3.95 | 7.30 | 1.26 | 16.34 | 1.03 | 11.19 | 12.53 | 1.642 |
| 8 | 4.14 | 2.18 | 8.11 | 1.25 | 16.62 | 1.07 | 9.63 | 13.03 | 2.641 |
| 9 | 3.36 | 2.94 | 8.63 | 1.30 | 15.25 | 1.04 | 6.42 | 12.63 | 1.874 |
| 10 | 3.67 | 2.56 | 7.76 | 1.21 | 15.73 | 1.28 | 7.97 | 13.14 | 3.300 |
| 11 | 3.71 | 2.95 | 5.90 | 1.17 | 14.34 | 1.18 | 7.45 | 10.46 | 2.101 |
| 12 | 3.46 | 2.03 | 6.21 | 1.19 | 14.26 | 1.14 | 7.12 | 11.33 | 2.430 |
| 13 | 2.89 | 3.31 | 9.75 | 1.22 | 15.72 | 1.43 | 4.42 | 14.12 | 2.918 |
| 14 | 2.54 | 1.36 | 7.22 | 1.52 | 15.42 | 1.11 | 9.53 | 14.03 | 1.959 |
| 15 | 2.40 | 1.21 | 7.50 | 1.02 | 16.65 | 1.04 | 9.42 | 12.24 | 2.030 |
| 16 | 2.47 | 1.79 | 6.82 | 1.11 | 15.27 | 1.35 | 5.40 | 11.01 | 3.052 |
| 17 | 1.98 | 2.61 | 6.55 | 1.25 | 15.86 | 1.11 | 4.58 | 11.35 | 1.991 |
| 18 | 2.51 | 1.16 | 6.70 | 1.86 | 15.66 | 1.01 | 4.54 | 13.02 | 2.402 |
| 19 | 1.82 | 3.25 | 5.23 | 1.60 | 14.58 | 1.40 | 3.32 | 10.46 | 2.976 |
| 20 | 2.00 | 1.38 | 6.88 | 1.90 | 15.49 | 1.12 | 3.53 | 11.31 | 2.235 |
| 21 | 1.70 | 0.63 | 7.82 | 1.52 | 15.12 | 1.08 | 2.51 | 12.77 | 2.034 |
| 22 | 1.44 | 0.74 | 5.52 | 1.16 | 14.34 | 1.33 | 1.98 | 9.64 | 2.749 |
